# Recent Advances in Root Canal Disinfection: A Review

**DOI:** 10.22037/iej.v12i4.17935

**Published:** 2017

**Authors:** Zahed Mohammadi, Hamid Jafarzadeh, Sousan Shalavi, Flavio Palazzi

**Affiliations:** a *Iranian Center for Endodontic Research, Research Institute of Dental Sciences, Dental School, Shahid Beheshti University of Medical Sciences, Tehran, Iran****; ***; b * Department of Endodontics, Faculty of Dentistry, Mashhad University of Medical Sciences, Mashhad, Iran; *; c *Private Practice, Hamedan, Iran;*; d *Department of Neuroscience, Reproductive and Odontostomatological Sciences, Federico II University of Naples, Naples, Italy*

**Keywords:** GentleWave Irrigation, Nano-Particles, Nano-Technology, Photodynamic Therapy, Photon-Induced Photo Acoustic Streaming, Root Canal Disinfection

## Abstract

The microbial biofilm is an important factor for human infection. Finding effective antimicrobial strategies should be considered for decreasing antimicrobial resistance and controlling the infectious diseases. Treatment of infected canal systems may not be able to remove all bacteria and so bacterial persistence after treatment may occur. Application of antibacterial nanoparticles may be a potential strategy to improve the elimination of bacteria from the canal. Furthermore, mechanism of action and applications of photodynamic therapy and Photon-induced photoacoustic streaming (PIPS) and GentleWave system was reviewed.

## Introduction

Different anatomy and complexities of the canal, in addition to dentin composition, are key challenges for effective disinfection in endodontics [1]. Antimicrobials such as sodium hypochlorite (NaOCl) are commonly used in endodontic treatment to combat microbial biofilms [2]; however, the anatomical complexities and undebrided parts of the canal may compromise their efficacy in endodontic treatment. 

In order to overcome the limitations of ordinary root canal irrigants and medicaments, using nanoparticles to disinfect the canal system has been proposed.

***Antibacterial nanoparticles (NPs)***

Nanomaterial denotes a natural or manufactured material containing unbound particles in which half or more of the particles in number and size is in the size range of 1-100 nm [3]. These materials present unique physicochemical properties, such as large surface area/mass ratio, and increased chemical reactivity [4, 5]. The increased number of atoms and increased surface to volume ratio compared with micro/macro-structures are suggested to contribute to the distinctly different properties of nanomaterials. These advantages may be exploited to design highly specific materials and devices to interact with at the subcellular and molecular level of the human body in order to achieve maximal therapeutic efficacy with minimal side effects [6, 7].

The electrostatic interaction between negatively charged bacterial cells and positively charged NPs, and also accumulation of increased number of NPs on the cell membrane of the bacteria have been associated with the loss of membrane permeability and unsuitable membrane function [8]. 

Antibacterial NPs show a broad spectrum of antimicrobial activity. According to Vier and Figueiredo [9, 10] metallic NPs of titanium, gold, zinc, and copper have attracted particular attention with different physical properties and spectra of antimicrobial effect. A study using MTT assay and confocal laser scanning microscopy demonstrated that 0.1% and 0.2% nanosilver gel is more effective on *Enterococcus faecalis* biofilm comparing camphorated phenol and chlorhexidine (CHX) gluconate [11]. An *in vitro* study showed that nanosilver gel is not efficient enough against *Enterococcus faecalis*; however, triple antibiotic paste and CHX gel showed better antibacterial activity than calcium hydroxide (CH) and so can be used as an alternative medicaments in endodontic treatment [12]. Zhang *et al.* [13] assessed the efficacy of CH with a silver NPs to eliminate the biofilm of *Enterococcus faecalis *and showed that silver NPs with CH has a significant inhibitory effect on the biofilm of *Enterococcus faecalis*. 

Barreras *et al.* [14] indicated that chitosan NPs acted synergistically with CHX through eliminating a greater amount of colony former units in both BHI-agar cultures and infected collagen membranes. Using CLSM and SEM analyses, Louwakul *et al.* [15] showed that CH NPs were more efficient than calcium oxide NPs in bacterial elimination in dentinal tubules. An *in vitro* study showed that adding silver NPs to MTA and CEM increased their antibacterial activity [16]. Fan *et al.* [17] investigated the substantivity of Ag-Ca-Si mesoporous NPs (Ag-MCSNs) on dentin and its antibacterial effects against *Enterococcus faecalis* and concluded that it may exhibit strong antibacterial activity against planktonic *Enterococcus faecalis* and better residual inhibition effects against *Enterococcus faecalis* growth on dentin than CH.

***Antimicrobial photodynamic therapy (APDT)***

APDT is a two-step procedure that involves the application of a photosensitizer, followed by light illumination of the sensitized tissues, which would generate a toxic photochemistry on target cells, leading to killing of microorganisms [18-20]. Nowadays, APDT is considered as a supplement to traditional protocols for canal disinfection. In an approach to adapt and improve the antimicrobial efficacy of APDT in endodontics, recent research has developed novel formulations of photosensitizers that displayed effective penetration into dentinal tubules, anatomical complexities, and antibiofilm properties. Well-designed clinical studies are currently warranted to examine the prospects for APDT in root canal disinfection [35, 36]. 

APDT may be combined with the usual mechanical instrumentation and chemical antimicrobials [21, 22]. Garcez *et al.* [23] compared the effectiveness of APDT, standard root canal therapy and the combined treatment to eliminate bacteria present in infected canals. Findings showed that root canal therapy alone reduced bacteria by 90% while APDT alone reduced it by 95%. The combination of two procedures reduced it by >98%. The bacterial regrowth observed 24 h after treatment was much more for either single treatment than the combination. In another study, Garcez *et al.* [24] evaluated the antimicrobial effect of APDT combined with root canal therapy in necrotic pulps infected with microflora resistant to a previous antibiotic therapy and concluded that endodontic treatment alone produced a significant decrease in numbers of microbial species, whereas the combination of endodontic treatment with APDT eliminated all drug-resistant species and surprisingly all teeth were bacteria-free. Garcez *et al.* [25] also showed that usage of APDT added to root canal therapy in canals infected with *Enterococcus faecalis* with the optical fiber is better than when the laser light is applied directed at the access cavity. 

Meire *et al.* [26] compared the antimicrobial efficacy of 2 high-power lasers (Er:YAG and Nd:YAG ) and 2 APDT systems with that of NaOCl action on *Enterococcus faecalis*. They concluded that NaOCl was the most effective in *Enterococcus faecalis* elimination, while Er:YAG laser also resulted in great decrease in viable counts. The use of both commercial APDT systems resulted in a weak reduction in the number of bacteria. The worth option was Nd:YAG irradiation.

According to George and Kishen [27, 28], APDT may destroy the functional integrity of bacterial cell walls, DNA, and membrane proteins of *Enterococcus faecalis*. The volume of damage on these targets is influenced by the photosensitizer solvent used during APDT. Soukos *et al.* [29] conducted APDT on a range of endodontic pathogens (methylene blue as photosensitizer) and reported complete removal of all bacteria except *Enterococcus faecalis* (53%). William *et al.* [30] measured antibacterial action of photoactivated disinfection (PAD) on *Peptostreptococcus micros*, *Streptococcus interme*dius, *Fusobacterium nucleatum* and *Prevotella intermedia*, and concluded that PAD killed these bacteria at statistically significant levels compared to controls.

Effect of PAD on bacterial endotoxins has also been studied. Endotoxin, a part of the cell wall of gram-negative bacteria, is composed of lipids, polysaccharides, and proteins and is referred to as lipopolysaccharide [31-33]. Shrestha *et al.* [34] evaluated the ability of APDT with chitosan-conjugated rose bengal NPs (CSRBnps) to inactivate endotoxins/LPSs. They concluded that photodynamically activated CSRBnps caused significant inactivation of endotoxins and the subsequent decrease of all tested inflammatory markers from macrophages. Antimicrobial CSRBnps in combination with APDT showed the potential to effectively inactivate endotoxins.

***Photon-induced photoacoustic streaming (PIPS)***

PIPS is based on the radial firing stripped tip with laser impulses of subablative energies of 20 mJ at 15 Hz for an average power of 0.3W at 50 μs impulses. These impulses induce interaction of water molecules with peak powers of 400W. This creates successive shock waves leading to formation of a powerful streaming of the antibacterial fluid located inside the canal, with no temperature rising [35, 36].

Unlike the conventional laser applications, the unique tapered PIPS tip is not mandatory to be placed inside the canal itself but rather in the pulp chamber only. This can reduce the need for using larger instruments to create larger canals so that irrigation solutions used during treatment can effectively reach to the apical part of the canal and also canal ramifications. This procedure can effectively remove both vital and nonvital tissues, kill bacteria, and disinfect dentin tubules [37, 38].

Peters *et al.* [39] showed that PIPS cannot completely remove bacteria from infected tubules but may remove biofilm better than passive ultrasonic irrigation. Jaramillo *et al.* [40] concluded that combinations of 20 s irradiation with Er:YAG laser *via* PIPS and 6% NaOCl has great effect in inhibiting* Enterococcus faecalis*. 

Ordinola *et al.* [41] evaluated the effect of PIPS using 6% NaOCl for the removal of an *in vitro* biofilm and showed an improved cleaning of the infected dentin on PIPS groups when compared to the PUI group. The extraordinary result from this study was the fact PIPS tip was placed 22 mm away from the target area, while sonic, ultrasonic, and passive irrigation were made at the exact target area. Jaramillo *et al.* [42] showed 83% disinfection of the conventional needle irrigation after 20 min of continuous irrigation *versus* 100% disinfection on PIPS, with a total of 1 min of irrigation with the same solution. Alshahrani *et al.* [43] also showed that the combination of PIPS+6% NaOCl is more effective than water+PIPS or just irrigation with 6% NaOCl. 

In an *in vitro* study, Zhu *et al.* [44] compared the antibacterial effect of PIPS versus a conventional irrigation. Findings revealed that there was no significant difference in CFU reduction and no bacteria could be observed by scanning electron microscopy in NaOCl, NaOCl+EDTA, and PIPS+NaOCl groups. Olivi *et al.* [45] showed that PIPS can increase the effect of irrigants commonly used in endodontic treatment such as NaOCl.

***Gentlewave irrigation***

Gentlewave (GW) (Sonendo, Laguna Hills, CA, USA) system aims to clean the root canal through generation of different physiochemical mechanisms including a broad spectrum of sound waves. Multisonic waves are initiated at the tip of GentleWave™ handpiece, which is positioned inside the pulp chamber [46]. It delivers a stream of treatment solution from the handpiece tip into the pulp chamber while excess fluid is simultaneously removed by the built-in vented suction through the handpiece. Upon initiation of flow through the treatment tip of the handpiece, the stream of the treatment fluid interacts with the stationary fluid inside the chamber creating a force which causes hydrodynamic cavitation. The continuous formation of microbubbles inside cavitation cloud generates acoustic field with broadband frequency spectrum that travels through the fluid into the entire canal [47]. 

According to Haapasalo *et al.* [47] the GW System provides tissue dissolution of eight and ten times faster than ultrasonic devices and needle irrigation, respectively. A study showed that GW system Gentle removed CH within 90 sec using water irrigation alone [48]. According to Molina *et al.* [49], the GW system showed greater cleaning and reduction in residual debris within the canals than those cleaned conventionally. The efficacy of GW system in removing separated instruments from the root canal has also been reported [50]. In a multi-center clinical study, Sigurdsson *et al.* [51] reported 97% successful healing in the teeth treated with the GW System at 12 months.

## Conclusion

Recent advances in root canal disinfection using new technology and on the basis of recent studies may improve the ability to disinfect the root canal system. However, conventional methods are still helpful for obtaining good prognosis.
